# Yohimbine-Induced Amygdala Activation in Pathological Gamblers: A Pilot Study

**DOI:** 10.1371/journal.pone.0031118

**Published:** 2012-02-02

**Authors:** Igor Elman, Lino Becerra, Evelyne Tschibelu, Rinah Yamamoto, Edward George, David Borsook

**Affiliations:** 1 Bedford Veterans Administration Medical Center and Department of Psychiatry, Cambridge Health Alliance, Harvard Medical School, Somerville, Massachusetts, United States of America; 2 P.A.I.N Group, McLean Hospital and Harvard Medical School, Belmont, Massachusetts, United States of America; 3 Clinical Psychopathology Laboratory, McLean Hospital and Harvard Medical School, Belmont, Massachusetts, United States of America; Centre for Addiction and Mental Health, Canada

## Abstract

**Rationale and Objectives:**

There is evidence that drug addiction is associated with increased physiological and psychological responses to stress. In this pilot functional magnetic resonance imaging (fMRI) study we assessed whether a prototype behavioral addiction, pathological gambling (PG), is likewise associated with an enhanced response to stress.

**Methods:**

We induced stress by injecting yohimbine (0.2–0.3 mg/kg, IV), an alpha-2 adrenoceptor antagonist that elicits stress-like physiological and psychological effects in humans and in laboratory animals, to four subjects with PG and to five non-gamblers mentally healthy control subjects. Their fMRI brain responses were assessed along with subjective stress and gambling urges ratings.

**Results:**

Voxelwise analyses of data sets from individual subjects, utilizing generalized linear model approach, revealed significant left amygdala activation in response to yohimbine across all PG subjects. This amygdala effect was not observed in the five control individuals. Yohimbine elicited subjective stress ratings in both groups with greater (albeit not statically significantly) average response in the PG subjects. On the other hand, yohimbine did not induce urges to gamble.

**Conclusions:**

The present data support the hypothesis of brain sensitization to pharmacologically-induced stress in PG.

## Introduction

As legalized gambling activities are rapidly expanding in our society so do gambling-related public health problems [1]. The overall lifetime prevalence of problem and/or pathological gambling (PG) in the general adult population is about 5% [2], [3] and its annual cost to the American society as a result of crime, decreased productivity and bankruptcies approximates $54 billion [4]. These figures likely underestimate the problems associated with PG because this is a more ‘silent’ addiction without characteristic symptoms of intoxication, needles' marks, or overdose, and therefore may only become apparent relatively late in the addiction process with the emergence of devastating and irreversible consequences, including attempted suicide in up to 24% of untreated individuals [4]–[6]. Hence, to improve diagnosis and treatment of PG it is important to identify its objective markers and their underlying neurobiology.

There is evidence that PG is associated with heightened stress responses. For example, gambling-related activities or exposure to gambling-related cues increases physiological stress responses like heart rate, skin conductance and norepinephrine concentrations in plasma and in cerebrospinal fluid [7]–[16]. There is also evidence that stress exposure causes gambling urges that may precipitate “relapse” to gambling [17]–[20]. Thus, like in drug addiction [21]–[23], stress can precipitate and exacerbate the maladaptive addictive behavior (gambling) and engagement in the addictive behavior or exposure to cues associated with maladaptive behavior (e.g., a slot machine) can lead to exaggerated or sensitized activation of the brain stress systems [20].

We have previously evaluated psychosocial stress levels in individuals with PG and found heightened scores across all measures; additionally, greater perceived severity and amount of daily stressors was associated with more urges to gamble [20]. Here, we further evaluated whether PG is associated with enhanced stress response by using yohimbine in conjunction with blood-oxygen-level dependent (BOLD) pharmacological magnetic resonance imaging (phMRI).

Yohimbine is an FDA-approved medication (oral formulation) for the treatment of male erectile dysfunction. It is a prototypical alpha-2 adrenoceptor antagonist that has been used in numerous studies to induce stress- and anxiety-like states in both humans and laboratory animals [24], [25]. In addition to its actions on the alpha-2 adrenergic systems, yohimbine also affects D2, alpha-1, 5HT1a, and benzodiazepine receptors [26]–[28]. However, termination of yohimbine's effects by alpha-2 agonists, clonidine and lofexidine, and replication of these effects by the selective alpha-2 adrenoceptor antagonist, RS-79948-197, renders non alpha-2 receptors'-related effects an unlikely mechanisms of yohimbine's stressogenic action [27], [28].

Due to the complexity of the brain and of the interactions among its various structures, it is possible on a theoretical basis to construct a lengthy list of brain regions, engaged by yohimbine-induced stress, activity in which could be altered in PG. In this study the amygdala was given *a priori* emphasis because blockade of presynaptic alpha-2 adrenoceptor leads to subjective stress responses [29] resulting from norepinephrine releases within the amygdala [30]–[32], which is also engaged by gambling cues reactivity [33] and by drug craving [34] in respective subjects with PG and with drug dependence. With these considerations in mind, it was hypothesized that, in comparison in healthy subjects, PG individuals would display amygdala hyperresponsivity to yohimbine.

## Methods

### Subjects

Participants of this pilot proof of concept/feasibility study comprised of four subjects [mean age (standard deviation, SD): 40.6 (13.0) years; 2 males and 2 females; 3 Caucasian and 1 African-American; weight: 80.4 (9.8) kg] meeting the Diagnostic and Statistical Manual of Mental Disorders, Fourth Edition, Text Revision (DSM-IV-TR [35]) criteria for PG and for no other Axis I DSM-IV-TR diagnosis. The control subjects were five mentally healthy individuals [age: 31.0 (9.3) years; 4 males and 1 female, 3 Caucasian and 2 African-American; weight: 74.5 (9.3) kg] who were free from any type of gambling problems. There were no significant group differences in age (t = 1.06; df = 7; p = 0.32), in weight (t = 0.93; df = 7; p = 0.32) and in gender distribution (p = 0.52; two-tailed Fisher's exact test).

The subjects were diagnosed using a best estimate format, involving all available sources of information, including clinical history, interview and the Structured Clinical Interview for DSM-IV [36], and the South Oaks Gambling Screen (SOGS [37]). This research was approved for safety and ethics by the Institutional Review Board of McLean Hospital. The participants were recruited by newspaper advertisement and underwent clinical assessments in return for a participation fee after providing a written informed consent to the McLean Hospital Institutional Review Board-approved protocol.

All subjects were in good physical health, without any history of head injury, loss of consciousness, brain tumor, seizures or cerebrovascular accident, as determined by neurological screening and by the Cornell Medical Index Health Questionnaire [38]. Recent drug and alcohol consumption was ruled out by negative results on urine toxicology screen and breathalyzer.

### Procedure

After completion of the clinical assessments and medical work up, the subjects reported for the procedure at the McLean Brain Imaging Center after having fasted and refrained from alcohol, tobacco, caffeine and physical activity for at least 10 h. One h before the imaging session, an intravenous catheter was placed into an antecubital vein for yohimbine infusion and was kept patent with a slow isotonic saline drip.

To date, numerous clinical studies employed intravenous yohimbine as a pharmacological stressor in neuroimaging and in clinical studies involving healthy subjects and psychiatric patients. The dose of yohimbine in these studies ranged between 0.125 mg/kg [39] to 0.4 mg/kg [40]–[43]. To explore potential dose-response relations, on the present study, two healthy controls were administered yohimbine at 0.3 mg/kg while the remainder of healthy controls and all PG subjects received yohimbine at 0.2 mg/kg.

Yohimbine was injected 5 min into a 30-min long scan in a single-blinded fashion. Four 2 ml yohimbine infusions were given over ten min (one control subject received the yohimbine infusion over 6 min), with each infusion lasting 20 sec. The onset of each infusion was separated by a 2-min interval. Continuous hemodynamic monitoring was performed and an advanced cardiac life support certified physician was present throughout the course of the study.

Analog scales for subjective ratings were projected via a LabView program and a back projection television system outside the Faraday shield of the scanner. The subjects rated their subjective measures of stress and gambling urges on a continuous Likert-type scale of 0 (none) to 12 (extreme). Ratings were initiated one min pre-infusion and collected once per min until 20 minutes post-infusion.

### Image acquisition

Scans were performed on a 3-Tesla Siemens Trio MR Imaging System (Siemens AG, Erlangen, Germany). A 3-plane scout scan (conventional FLASH sequence with isotropic voxels of 2.8 mm) was acquired. This was used for prescription of the fMRI image stack (gradient echo planar imaging [EPI], repetition time/echo time = 2000/30 msec, 220 mm×220 mm field of view [FOV], 30 3-mm coronal slices starting from the anterior pole, no gap, right-left readout, 64×64 pixel, full k-space acquisition, no sensitivity encoding [SENSE] acceleration; pulse sequence-enhanced version of the Siemens epibold; total acquisition time 9×4: 04 min). Automatic second-order shimming was performed over the fMRI imaging volume before acquisition. After the functional scans, subjects had a conventional T1 scan performed on the same functional prescription and therefore with identical susceptibility distortion (68 T1 weighted coronal slices, FOV = 220 mm×220 mm, 256×256 pixel, 3-mm thick were acquired covering the whole brain, “matched warped”) [44] and a standard T1 weighted magnetization prepared rapid gradient echo (MPRAGE) three-dimensional (FOV = 256 mm×256 mm×170 mm, 256×256×128) for anatomic segmentation and parcellation.

### Image analysis

The image analyses were conducted after the completion of data collection from all participants. Functional MRI images were aligned to the high-resolution T1 weighted MPRAGE images in two steps with FMRIB's Linear Image Registration Tool (FLIRT). Instead of using a conventional high-signal-to-noise-ratio EPI image for the intermediate, we employ a high-resolution, T1-weighted “match-warped” EPI image that increases the precision of the alignment between the functional dataset and the high-resolution MPRAGE [44]. The MPRAGE was then aligned to Montreal Neurological Institute (MNI) space with FLIRT.

Data processing was performed with FSL release 4.0 (FMRIB Analysis Group, Oxford University, United Kingdom; http://www.fmrib.ox.ac.uk.ezp-prod1.hul.harvard.edu/fsl/), specifically FEAT version number 5.92. Preprocessing procedures included the following steps. First, image spikes were detected by FSL's outlier detection program (fsl_motion_outliers) and used as covariates of no interest in the statistical analysis. Second, all images within a scan were aligned to image #230 (in the middle), with mcflirt [45], with 6 degrees of freedom. If the maximum Euclidean deviation from this reference exceeded 3.0 mm (the smaller voxel dimension), the scan was discarded. Third, non-brain tissue was removed. Fourth, spatial filtering was performed, with a Gaussian kernel with 5-mm full width half maximum. Finally, global normalization was performed, such that the average over all voxels and images was fixed at 10000.

### phMRI infusion model (Figure 1)

**Figure 1:**
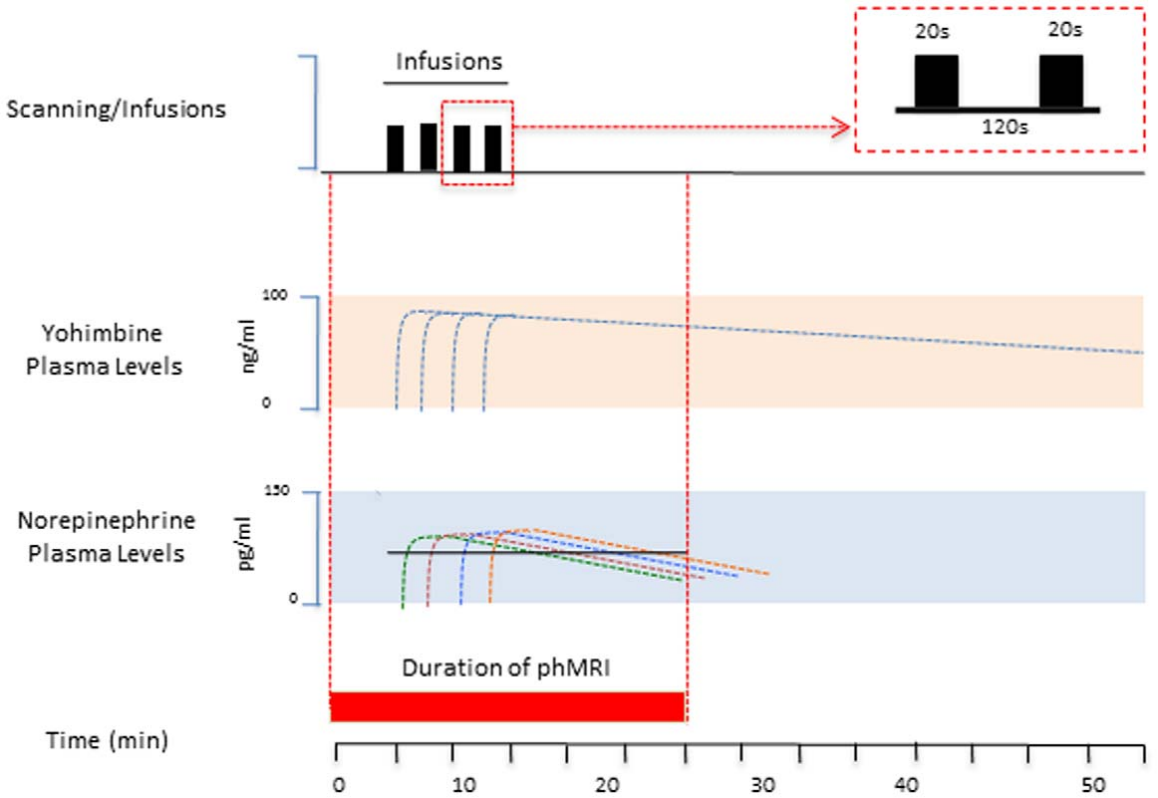
phMRI of Yohimbine. **Top: Yohimbine Infusion Procedure.** Following a 5 min baseline, yohimbine was infused in four separate infusions, each infusion lasting 20 sec, and with an interinfusion interval of 2 min, as shown in the box (see Text). **Middle: Plasma levels of Yohimbine.** Based on prior reports [77] doses of yohimbine reach maximal levels immediately after intravenous infusion and remain high for at least 20 min. **Bottom:** Plasma levels of norepinephrine rapidly follow yohimbine infusions [78]. These levels also remain high for the duration of the phMRI data acquisition.

Based on a prior report in the literature, following intravenous infusion of yohimbine (0.25–0.5 mg/kg) in healthy subjects, the distribution is rapid, the elimination half-life is 0.25–2.5 h and norepinephrine increases 3-fold within 15 minutes [46].

Voxelwise data analyses were carried out in the similar fashion to the previously reported by our group on an phMRI study [47]. Statistical maps were generated using a generalized linear model approach with two possible responses of interest: a gradual increase in activation (Ramp) and a rapid onset of activation followed by a gradual decay (Decay). An additional response of interest was generated through appropriate contrasts to obtain a step-wise response once the injection of yohimbine begins. The full model also included motion parameters estimates and spikes' temporal appearance as covariates of no interest.

We used a mixture of Gaussian distributions, the number and parameters of each were optimally determined. Statistical thresholds were determined utilizing a mixture model approach [48], [49]. Each voxel had 3 probabilities assigned to belong to one of these classes: deactivation, null hypothesis, and activation class. Each voxel had its p-value corrected, that is to say, thresholded so that the posterior probability associated with the activation class was 0.5 or larger. For standard presentation, statistical maps were transformed into the MNI-standard brain space. The amygdala was identified from the Oxford-Harvard probabilistic atlas issued with FSL's software package.

## Results

### Subjective ratings

Mean baseline subjective stress (i.e., distress) rating was 0 for pathological gamblers and 0.2 (1.1) for healthy controls, respectively. Throughout the 20 min following yohimbine administration, PG and healthy subjects demonstrated respective increases in subjective stress ratings to 4.0 (5.4) and 2.4 (2.1) thus yielding mean change from the baseline (peak value minus baseline value) of 4.0 (5.4) and 2 (2.2). The mean group difference in the stress ratings' changes was 1.8 with pooled SD of 3.4, corresponding to a medium effect size of d = 0.53 SD. To achieve 80% power to detect stress ratings differences of this magnitude at p<0.05 would require n = 45 subjects per cell.

To determine effects of yohimbine on stress self-ratings, a one-way analysis of variance (ANOVA) with a repeated-measures design was conducted. Diagnosis (PG and healthy subjects) was the grouping factor and Drug (pre- and post yohimbine) was the within-subjects factor. This ANOVA resulted in a significant drug effect (F = 5.83; df = 1; p<0.05) but no significant group effect (F = 0.25; df = 1; p = 0.63) or drug by group interaction (F = 0.53; df = 1,7; p = 0.49). Repeating the latter analysis after excluding the two subjects who received 0.3 mg/kg of yohimbine (both in the control group) revealed a trend for significant drug effect (F = 4.30; df = 1, 7; p<0.09), which would have been statistically significant had this result been predicted *a priori*.

Yohimbine produced no meaningful effect on subjective self-reports of gambling urges in PG subjects [pre- and post-yohimbine values: 1.3 (1.0) and 1.0 (0.8)].

### Voxelwise analyses of data sets from individual subjects

**Figure 2:**
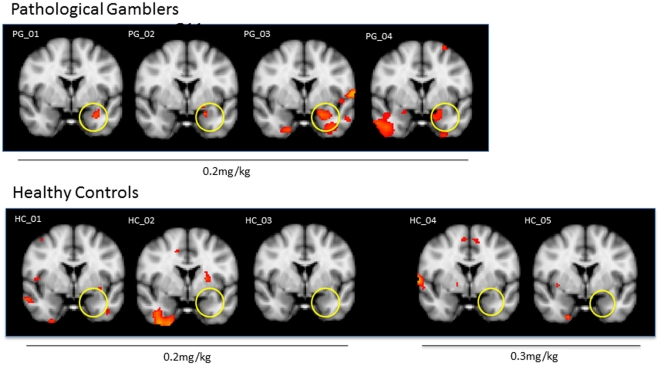
Individual data set. Clusters of activation (colored; corrected p<0.001) from voxelwise analyses of the effects of yohimbine infusion obtained from individual subjects projected onto a background (grayscale) representing subjects' mean high-resolution anatomic image. Coordinates of coronal slices are in accordance with the Montreal Neurological Institute (MNI) space. Please note: two healthy controls received yohimbine at 0.3 mg/kg; the rest of the subjects were administered 0.2 mg/kg. PG-pathological gamblers; HC-healthy controls.

The MNI x, y, and z coordinates of the peak voxel in the left amygdala cluster of activation (corrected p<0.001) along with the cluster volume for each PG subject are presented in **Table 1**. After an infusion of yohimbine (0.2 mg/kg), left amygdala activation was observed for each subject diagnosed with PG (**Figure 2**). This is indicated by significant BOLD responses that correspond to a gradual increase in activation. The other models of a rapid onset and decay or a step-wise response were not apparent. Healthy controls did not display significant amygdala activation at the same dose and even at the 0.3 mg/kg dose (n = 2). No significant deactivation was observed at the individual level for the two groups. The two-tailed Fisher's exact test, performed to compare the proportion of subjects with yohimbine-induced amygdala activation (4/4 vs. 0/5), revealed a significant group difference (p = 0.008).

**Table 1:** Individual clusters of amygdala activation in pathological gamblers (corrected p<0.001).

	Coordinates (mm)			Z value	Volume (mm^3^)
	X	Y	Z		
**PG_01**	−18	−6	−20	1.23	304
**PG_02**	−22	0	−18	1.13	112
**PG_03**	−22	−8	−14	1.32	376
**PG_04**	−22	2	−18	1.22	496

The coordinates are taken from the MNI Brain Atlas. The origin of the coordinates refers to the anterior commissure.

Other brain regions that were significantly activated in individual PG subjects included right frontal pole and left hippocampus. The following areas were significantly activated in single healthy control subjects: left putamen, left caudate, right pallidum, left anterior cingulate cortex and left frontal pole. None of these areas of activations overlapped across the subjects.

## Discussion

The major finding of this study was that pathological gamblers had significantly greater yohimbine-induced activations in the left amygdala region. A similar approach in substance use disorders indicate that drug- and alcohol dependent patients are hypersensitive to yohibine-induced stress as evidenced by their startle hyperreflexia [40], [50], their elevated stress hormones [40], [51] and the emergence of withdrawal-like symptomatology [41].

The present results extend our early work with psychometric assessments in which we found increases in stress and negative mood states in PG subjects [20]. Our data with yohimbine, an alpha-2 adrenoceptor antagonist that activates both peripheral and central noradrenergic systems [25], are also consistent with prior reports of sympathetic hyperresponsivity in PG [11], [12]. Together, our current and previous results suggest that PG is associated with heightened reactivity to various types of stress and that the left amygdala is involved in this association.

Another main finding in our study is that the enhanced fMRI response to yohimbine in PG subjects was only observed in the left amygdala. This laterality in response to stress may be consistent with previous findings. For example, a systematic review of 54 fMRI and positron emission tomography studies suggests that the left amygdala is more likely to be activated than the right amygdala during emotional tasks [52]. The procedure employed here, however, does not allow firm separation of physiological and emotional stress components involved in pathological gamblers' amygdala activations. Answering this question would require an exclusively emotional task that does not employ pharmacological agents.

### Methodological considerations

Under the current experimental conditions, yohimbine was an effective stressor eliciting stress responses in both groups. The failure to detect significant group differences in the subjective stress reports may be secondary to the small sample size, that is to say, the Type II error. Also, given the greater variability in stress ratings in the PG group, in a larger sample the stress response could be correlated with the change in BOLD signal in the amygdala. On the other hand, some of the stress' aspects may be subliminal and not readily amenable for self-reports. For instance, on a recent study, alcohol dependent patients showed heightened hypothalamic-pituitary-adrenal axis' responses to alcohol cues and to a generic stressor, but only the former and not to the latter type of the stimulus elevated subjective stress ratings [53].

Another caveat that should be considered in interpreting these data pertains to the age confound as PG patients averaged 10 years older than healthy subjects. This is an unlikely confound as there is a considerable amount of clinical data suggesting opposite to our finding age-related declines in the amygdala function [54]–[56]. Nonetheless, a study of an age-matched control group may be warranted. Similar caveat is pertinent to the dose yohimbine as two controls received a higher dose of 0.3 mg/kg even though given the lack of amygdala activations in healthy people exposed to 0.3 mg/kg of yohimbine, a compelling *a fortiori* argument could be that such a response will be also absent with 0.2 mg/kg. These and other important factors that were not a part of the present study design (e.g., plasma yohimbine concentration) may need to be assessed in future studies.

Contrary to the prior reports on yohimbine-induced craving in opioid- [41] and alcohol- [57] dependent subjects, participants in the current experiment reported no meaningful change in the level of their gambling urges. It has been previously proposed that gambling urges and drug craving are analogous phenomena [58]–[60], but our findings perhaps suggest otherwise. More yohimbine studies, matching drug dependent patients and pathological gamblers by the severity of their addiction, may be needed to ascertain potential gambling urges insensitivity to noradrenergic stimulation. On the other hand, an important factor, not addressed by the present design, which could yield information bearing upon elucidation of gambling urges' triggers, is the expectancy context. Thus, the fMRI scanner or the laboratory context may mask stress-related gambling urges while gambling cues or knowledge that gambling opportunities are available during or after the infusion could produce the opposite effect [61].

A limitation with this study's design is its inability to resolve the risk factor versus acquired origin of the hyperresponsive circuitry in PG. While it is tempting to speculate that similarly to drugs of abuse [21], [62]–[64] gambling activities contribute to the brain stress sensitization, an alternative interpretation is also plausible. Thus, persons with high stress sensitivity (potentially due to prior stressful experiences) may have greater propensity for development of gambling addiction owing to stress-related neuroadaptations in the dopaminergic circuits [65]. Regardless of the origin, a feedforward interaction could be a possible outcome wherein gambling and gambling-related cues trigger stress responses while stress promotes gambling.

### Concluding remarks

The present findings together with sensitized brain metabolic reactions to gambling-related stimuli [66], [67] are reminiscent of a cross-sensitization phenomenon observed in substance use disorders. The latter term typically refers to a situation where prior exposure to one stimulus (e.g., drug) increases subsequent response to itself [68]–[71] and to a different stimulus (e.g., stress) [62]–[64] and in the reversed order, enhancement of drug motivational states e.g., craving [72], [73] following prior stress exposure [65]. Indeed, the sensitized stress responses in PG are mostly conspicuous in the context of gambling and gambling-related cues [7]–[12], whereas stress is a key factor responsible for gambling urges [20] inherent in the chronically relapsing nature of PG [17]–[19]. This raises the possibility of overlapping and sensitized neuropsychobiological systems engaged by stress- and gambling-related stimuli in patients with PG. Such a testable hypothesis could be evaluated in PG subjects by juxtaposing responses to functional neuroimaging probes that reliably activate both brain stress and motivational circuits.

Finally, it is noteworthy that stress-related noradrenergic system has been implicated in the pathophysiology of impulsivity at large [74] not just in PG, which is while considered by some to be a prototype non pharmacological addiction [75], [76] is actually classified as an impulse control disorder [29]. Therefore it would also be of interest to test whether the observed stress alterations are generalizable to impulse control disorders as a class.
